# Cancer and Exercise: Warburg Hypothesis, Tumour Metabolism and High-Intensity Anaerobic Exercise

**DOI:** 10.3390/sports6010010

**Published:** 2018-01-31

**Authors:** Peter Hofmann

**Affiliations:** Institute of Sports Sciences, Exercise Physiology, Training & Training Therapy Research Group, University of Graz, Max Mell Allee 11, Graz 8010, Austria; peter.hofmann@uni-graz.at; Tel.: +43-316-380-3903

**Keywords:** high-intensity exercise, cancer metabolism, lactate, inhibition, exercise prescription

## Abstract

There is ample evidence that regular moderate to vigorous aerobic physical activity is related to a reduced risk for various forms of cancer to suggest a causal relationship. Exercise is associated with positive changes in fitness, body composition, and physical functioning as well as in patient-reported outcomes such as fatigue, sleep quality, or health-related quality of life. Emerging evidence indicates that exercise may also be directly linked to the control of tumour biology through direct effects on tumour-intrinsic factors. Beside a multitude of effects of exercise on the human body, one underscored effect of exercise training is to target the specific metabolism of tumour cells, namely the Warburg-type highly glycolytic metabolism. Tumour metabolism as well as the tumour–host interaction may be selectively influenced by single bouts as well as regularly applied exercise, dependent on exercise intensity, duration, frequency and mode. High-intensity anaerobic exercise was shown to inhibit glycolysis and some studies in animals showed that effects on tumour growth might be stronger compared with moderate-intensity aerobic exercise. High-intensity exercise was shown to be safe in patients; however, it has to be applied carefully with an individualized prescription of exercise.

## 1. Introduction

The role of physical activity (PA) and exercise training has been extensively investigated in the entire cancer continuum from prevention to treatment and post-treatment applications [1,2,3,4,5], and guidelines for cancer survivors have been presented [6,7]. There is ample evidence suggesting regular physical activity to be related to a reduced risk for various forms of cancer [2,3]. Regular moderate to vigorous physical activity (any PA of an intensity >3 metabolic equivalents (METs)) was shown to be associated with a lower risk for developing several cancers, including proximal (24%) and distal (23%) colon, endometrial (17%, albeit only in postmenopausal overweight/obese women), breast (12%), prostate (10%), gastroesophageal (18%), ovarian (11%), renal (12%), lung (24%), and pancreatic (11%) cancer [3]. In this meta-study, Moore et al. presented data on a total of 1.44 million participants, which included 186,932 cancers. Compared to low levels, high levels of leisure-time physical activity were associated with lower risk in 13 different cancer types. These authors showed reduced risk for oesophageal adenocarcinoma (hazard ratio (HR), 0.58; 95% confidence interval (CI), 0.37–0.89), liver (HR, 0.73; 95% CI, 0.55–0.98), lung (HR, 0.74; 95% CI, 0.71–0.77), kidney (HR, 0.77; 95% CI, 0.70–0.85), gastric cardia (HR, 0.78; 95% CI, 0.64–0.95), endometrial (HR, 0.79; 95% CI, 0.68–0.92), myeloid leukaemia (HR, 0.80; 95% CI, 0.70–0.92), myeloma (HR, 0.83; 95% CI, 0.72–0.95), colon (HR, 0.84; 95% CI, 0.77–0.91), head and neck (HR, 0.85; 95% CI, 0.78–0.93), rectal (HR, 0.87; 95% CI, 0.80–0.95), bladder (HR, 0.87; 95% CI, 0.82–0.92), and breast cancers (HR, 0.90; 95% CI, 0.87–0.93). When adjusted for body mass index, associations were modestly attenuated for several cancers, but 10 of 13 inverse associations remained statistically significant. Leisure-time physical activity was associated with higher risks of malignant melanoma (HR, 1.27; 95% CI, 1.16–1.40) and prostate cancer (HR, 1.05; 95% CI, 1.03–1.08), which were independent of body mass. Smoking status modified the association for lung cancer but not for other smoking-related cancers [3]. Findings from 71 prospective cohort studies also showed a dose-response effect of PA on cancer mortality. Individuals with the highest physical activity had a HR of 0.83 (95% CI 0.79 to 0.87) and 0.78 (95% CI 0.74 to 0.84) for cancer mortality in the general population and among cancer survivors, respectively. There was an inverse non-linear dose response between the effects of physical activity and cancer mortality. A minimum of 2.5 h/week of moderate-intensity activity significantly reduced cancer mortality by 13% in the general population. Fifteen MET-h/week of physical activity reduced cancer mortality risk by 27%. An even greater protective effect occurred in cancer survivors undertaking physical activity post-diagnosis versus pre-diagnosis, and the same amount of physical activity per week decreased the risk by 35% and 21%, respectively [8]. In an earlier study, Arem et al. (2015) presented a pooled analysis of the dose-response relationship between leisure time PA and mortality. Data from six studies and a total of 661,137 men and women and 116,686 deaths were included for a median follow-up time of 14.2 years. Compared to individuals reporting no leisure-time physical activity, these authors observed a 20% lower mortality risk among those performing less than the recommended minimum of 7.5 MET/h per week (HR, 0.80 (95% CI, 0.78–0.82)), a 31% lower risk at 1–2 times the recommended minimum (HR, 0.69 (95% CI, 0.67–0.70)), and a 37% lower risk at 2–3 times the minimum (HR, 0.63 (95% CI, 0.62–0.65)). The authors suggested an upper threshold for mortality benefit at 3–5 times the physical activity recommendation (HR, 0.61 (95% CI, 0.59–0.62)) but compared to the recommended minimum, the additional benefit was modest (31% vs. 39%). Importantly, there was no evidence of harm at 10 or more times the recommended minimum (HR, 0.69 (95% CI, 0.59–0.78)) [9]. A recent systematic review on the dose-response relationship between PA and the risk of breast cancer, colon cancer and cardiovascular diseases identified 174 relevant articles included in this meta-study. Higher levels of total physical activity were significantly associated with lower risk for all outcomes, but major gains occurred already with lower levels of activity (up to 3000–4000 MET min/week). Compared with insufficiently active individuals (total activity <600 MET min/week), the risk reduction for those in the highly active category (≥8000 MET min/week) was 14% (RR 0.863, 95% CI 0.829 to 0.900) for breast cancer and 21% (0.789, 0.735 to 0.850) for colon cancer. People who achieved total physical activity levels several times higher than the current recommended minimum level had a significantly reduced risk for breast and colon cancer and cardiovascular diseases [10].

Summarizing these actual studies provide sufficient evidence about dose-dependent beneficial associations between physical activity and cancer risk to suggest a causal relationship [3,4,5].

However, the main mechanisms of risk reduction are yet to be discovered. Exercise, which is the structured mode of physical activity with defined intensity, duration, and frequency, is generally associated with positive changes in fitness, body composition, and physical functioning as well as in patient-reported outcomes such as fatigue, sleep quality, or health-related quality of life in general [11]. However, emerging evidence indicates that exercise may also be directly linked to the control of tumour biology [5]. McTiernan [12] suggested physical activity to be linked to cancer protection through exercise-dependent reductions in cancer risk factors, such as sex hormones, insulin/insulin like growth factor (IGF), and inflammatory markers. In their recent review, Hojman et al. [5] suggested that these effects of regular exercise decrease the risk of cancer and can control tumour growth independent of the specific cancer diagnoses. Overall, long term training may decrease systemic levels of risk factors such as shown for breast cancer. However, these effects were modest and closely related to weight loss. The authors suggested therefore that controlling caloric intake may be a more feasible approach for lowering the systemic levels of risk factors such as sex steroid hormones, insulin, and inflammatory markers. These tumour growth–inhibitory effects may be mediated by several different mechanisms such as direct effects of exercise on tumour-intrinsic factors, tumour growth kinetics and formation, tumour metabolism, the immunological profile of the tumour, exercise-dependent tumour–organ crosstalk and immunological control of tumour growth, and muscle-to-tumour crosstalk. However, their individual contributions to the inhibitory effect of exercise may vary in importance across different cancer diagnoses. These authors also highlighted that exercise may improve the efficacy of anti-cancer therapy [5,13]. In murine studies, it was shown that exercise training controls cancer progression through direct effects on tumour-intrinsic factors, such as growth rate, metastasis, and tumour metabolism and immunogenicity, regulates tumour growth dependent on systemic factors and reduces adverse effects shown in detail in the review by Hojman et al. [5].

Beside a multitude of effects of exercise on the human body, one underscored possible effect of exercise training is to target the specific metabolism of tumour cells, namely the Warburg-type highly glycolytic metabolism [14,15]. Tumour metabolism as well as the tumour host interaction may be selectively influenced by exercise training, dependent on exercise intensity, duration, frequency, and mode [16]. It was suggested that exercise might be able to reprogram the distant tissue microenvironment not directly involved in the exercise response. Beside the chronic effects of physical activity and exercise training, Dethlefsen et al. [17] highlighted the impact of acute bouts of exercise inducing alterations that in magnitude by far surpass the adaptations seen with long-term training. Continuous boluses of exercise factors are suggested to have the potential to impact cancer cell biology and viability as shown for breast cancer [17]. Studies in other diagnoses add to this effect, showing reduced cancer growth by exercise-mediated increases in immune cells, epinephrine, and muscle derived factors [18]. Pre-clinical studies suggest that systemic changes during exercise may directly inhibit breast cancer cell progression, and the authors point out that these acute exercise-induced changes in classical risk factors are minor and therefore not plausible candidates for the protective effect. In contrast, major increases in the levels of myokines and catecholamines are suggested to have the potential to directly regulate tumour growth [17]. These authors mention that several other circulating metabolites (e.g., lactate, pyruvate, succinate, and malate) and exosomes could potentially also affect cancer progression. With respect to these acute effects an individualized prescription of exercise was therefore argued to be important but has not been addressed properly in recent research [19].

Despite the complexity of the multitude of influencing factors prescribed in detail elsewhere [5,12,13,17,18], the present review focuses solely on the specificity of tumour metabolism and the designated knowledge about glucose and lactate metabolism during exercise training known as the Lactate Shuttle Theory [20,21]. This theory has also been prescribed for tumour tissue [22] and tumour–host interaction [23]. A high glycolytic activity of most cancer types is one of the hallmarks of cancer [24,25], which has been suggested as a sweet-tooth [26,27]. This weak point may be an attractive target as a therapy option [28]. Recently, Ruiz-Casado et al. [29] presented a review about the relationship between exercise and the hallmarks of cancer from mechanistic studies. They summarized the effects of exercise on sustaining proliferative signalling, evading growth suppressors, resisting cell death, enabling replicate immortality, inducing angiogenesis, activating invasion and metastasis, tumour-promoting inflammation, reprogramming of energy metabolism, and evading immune destruction, which are accepted traits of cancer cells. The authors concluded that more mechanistic studies are needed to identify the molecular mediators and signalling pathways that may help to explain how regular exercise can affect these various hallmarks of cancer [24]. Increasing evidence supports the potential anti-tumorigenic effects of a physically active lifestyle, promoting apoptosis in tumour cells or innate immunity against some cancers. Critically, Ruiz-Casado et al. [29] mentioned that no study has yet addressed whether exercise affects genomic instability and the heterogeneity among studies in both exercise and cancer models. As a major limitation, these authors mention that the potential anti-tumorigenic effects of exercise have usually been demonstrated with spontaneous wheel running, forced mouse treadmill running, or swimming. While wheel running does not allow establishing a predetermined training load but being a more natural form of exercise such as physical activity, forced treadmill running would be more a model of regulated aerobic exercise training [29].

Most studies apply moderate-to-vigorous aerobic-type exercise, which has been recommended and applied in patients. This mode of exercise was shown to be safe and effective [7,19]. However, for some types of cancer, the American College of Sports Medicine roundtable on exercise guidelines for cancer survivors [7] explicitly recommended low intensity instead of moderate to vigorous. Although there is still fear about high-intensity exercise, it was shown that this type of exercise gains benefits different from low- and moderate-intensity exercise, though the mechanisms remain unclear [30]. Ruiz-Casado et al. [29] highlighted that elite athletes exposed to strenuous exercise for several years had a 40% lower risk of overall cancer mortality than the general population supporting a dose-response effect.

## 2. Aerobic Glycolysis as a Main Metabolism of Several Tumours

One outstanding feature of cancer is a markedly different metabolism compared with normal cells. This specification of metabolism has been described recently as a main possible target (sweet tooth) for cancer therapy [26,27,31,32,33,34,35,36], applying the so-called Warburg hypothesis presented around 80 years ago [14,15,30]. In contrast to normal tissues, it was shown by Warburg in 1927 [14] that cancerous tumours frequently develop a modified glucose metabolism whereby a significant portion of the blood glucose consumed by the tumour is converted one step beyond pyruvate to lactate acid, even when oxygen is plentiful—the so-called “Warburg effect” [27,28,37]. Even when the tumour mitochondria are sufficiently supplied with oxygen to metabolize all the pyruvate formed from the blood glucose to carbon dioxide and water, the tumour converts glucose to lactic acid to produce energy (ATP) via glycolysis. As glycolysis produces only two net molecules of adenosine_triphosphate (ATP) per glucose molecule converted to lactic acid, the cancer cells remodel their glycolytic and mitochondrial metabolism such that the former is upregulated and the latter is downregulated. In summary, the “Warburg effect” refers to cancerous tumours’ increased utilization of the glycolytic pathway for energy production and to produce intermediates for increased cell growth and division [25], even though plenty of oxygen is available to support the mitochondrial function [31]. Importantly, almost all cancer types present this predominant glycolytic metabolism, highlighting a general cancer type of metabolism [30,38]. Hirschhaeuser et al. [39] named lactate a metabolic key player in cancer and prescribed that the metabolic switch contributes to immune escape, cell migration, and radio-resistance. The Warburg Effect is fully accepted as a hallmark of cancer, but it was critically mentioned that the study of cancer cell metabolism was diverted when investigators began to employ genomic techniques to better understand cancer biology [39]. These authors pointed out a lack of understanding about the meaning and role of the Warburg Effect in cancer, which did not progress in parallel and may have impeded a full comprehension of cancer biology. Consequently, there is a lack of understanding of the roles of lactate in promoting carcinogenesis and tumorigenesis. Today, there is a resumption of interest in understanding the role of lactate in cancer, and there is growing significance for the role of lactate in normal physiology and its use in the treatment of injuries and illnesses [40,41,42]. For a long time, lactate was viewed as a waste product of anaerobic metabolism, but lactate is now recognized an important energy fuel, a major gluconeogenic precursor, and a highly active signalling molecule with hormone-like properties [40]. San-Millan and Brooks [40] view cancer cell biology from perspective of the lactate shuttle concept [20,21] where lactate produced at one intracellular site was prescribed to induce autocrine, paracrine, and endocrine responses. These authors there compared metabolic characteristics common between working muscles and cancer cells, and they thought new knowledge in skeletal muscle exercise metabolism reasonable for application by cancer biology researchers. They mentioned the hope that this new perspective would lead to a wide discussion about lactate metabolism in cancer to develop new diagnoses and therapeutics.

Recently, Semenza [43] argued that tumours are not exclusively hypoxic but also contain well-oxygenated (aerobic) regions. Aerobic cancer cells can take up lactate via the lactate transporter monocarboxylate transporter 1 (MCT1) and utilize this substrate for oxidative phosphorylation [44] such as is well-known for muscle and other tissue metabolism [20,21,22,45,46,47]. Semenza [43] showed that by the inhibition of the MCT1, aerobic cancer cells take up glucose rather than lactate and force hypoxic cancer cells to die due to glucose deprivation, offering an interesting therapeutic approach. It was shown that MCT1 expression was detected exclusively in non-hypoxic regions of human cancer biopsy samples, and this suggested that MCT1 inhibition holds potential as a novel cancer therapy [43,48,49]. This specific metabolism of hypoxic cancer cells leads to a marked extra-cellular acidosis due to both increased H^+^ production and increased H^+^ efflux through hypoxia-inducible factor 1 (HIF-1) mediated trans-activation of transporters such as carboanhydrase IX, monocarboxylic transporter 4 (MCT4) and Na^+^/H^+^ exchanger 1 (NHE1) [43].

Sonveaux et al. [44] demonstrated the existence of some kind of metabolic symbiosis between hypoxic and aerobic cancer cells, in which lactate produced by hypoxic cells is taken up by aerobic cells, which use it as their principal substrate for oxidative phosphorylation. At the molecular level, MCT1 was recognized a key player in this symbiotic relationship. Contrary to MCT4, the expression of MCT1 is hypoxia-repressed rather than hypoxia-induced, and MCT1 transports lactate into, rather than out of, cancer cells, which is also well described for skeletal muscle and other tissues’ lactate shuttles [20,21,22]. The oxygen-dependent expression of MCT1 allows aerobic cancer cells to efficiently take up lactate and utilize lactate as an energy substrate, thereby freeing these cells from the need to take up large quantities of glucose [42]. Interestingly, Semenza [43] pointed out that the well-known recycling of lactate in exercising muscle directed him to the existence of this symbiotic relationship between aerobic and hypoxic cancer cells. Just as tumours co-opt physiological mechanisms regulating vascularisation, which are orchestrated by the hypoxia-inducible factor 1 (HIF-1), so do they modulate metabolism through a programme that functions to efficiently distribute glucose and lactate to fast-twitch glycolytic and slow-twitch oxidative muscle fibres described in the lactate shuttle theory [20,21,45,46,47].

Although the glycolytic phenotype was described as a prominent indicator of the tumour metabolism, the real situation is far more complex insofar as a cancer cell that is hypoxic at one moment may be aerobic an hour later and vice-versa, suggesting a cyclic variation in oxygenation. This, in turn, implies a dynamic regulation of the metabolic symbiosis, such that cells may cycle between lactate-producing and lactate-consuming states [21], suggesting the interventions targeted to glycolysis are questionable [50]. However, recent results highlight some real possibilities to positively influence tumour metabolism, which offers a new therapeutic option [37].

One cornerstone to understand the effects shown in recent exercise training studies may therefore be lactate metabolism in every single exercise bout [13,51]. It is a well-known effect in healthy athletes that elevated systemic lactate levels during repeated high-intensity anaerobic exercise inhibit glycolysis and net lactate production [52]. Recently, we show that this principle also works with high-intensity anaerobic exercise preceding a subsequent intense exercise bout [53]. Thus, one may hypothesize that high-intensity anaerobic exercise may counteract tumour metabolism by inducing a systemic acidosis, inhibiting tumour cell glycolysis, and thus reversing the Warburg phenotype metabolism of cancer cells as proposed from a model simulation [54]. Repeated boluses of potent anti-cancer factors can drive the decrease in cancer onset and recurrence by their cumulative effects. Thus, each bout is suggested to provide a small reduction in cancer cell growth. However, if repeated numerous times a week for several months, a substantial anti-oncogenic effect may be expected [13]. These authors point out that it might be more important to establish the most potent acute systemic response, rather than designing training interventions aiming on weight loss [17]. In this study lactate significantly increased six-fold, epinephrine 2.9-fold, and nor-epinephrine 2.2-fold. No changes were found for insulin levels, but systemic concentrations of IL-6 (2.1-fold), IL-8 (20%) and TNF-α (13%) were detected while IL-10 was unchanged [17].

## 3. Glycolysis Inhibition as a Possible Therapy Target of Tumour Metabolism

Several authors recognized that lactate metabolism is crucial for malignance and may therefore be a target for drug design [25,28,48,49,55,56,57,58,59,60]. Lactate metabolism was shown to be directly correlated with the prognosis of cancer. Accelerated glucose consumption is associated with tumorigenesis, and it is hypothesized that the reversal of this metabolic phenotype—the Warburg effect—would impair tumour growth and viability. However, the tumour glycolytic phenotype enhances lactate production by these cells, and this acidifies the environment of the tumour. Moreover, lactate produced by tumour cells is known to inhibit T-cell responses and thus impairs immunological pathways that could regulate tumour growth [61]. Tumour lactate production has been pointed out as a potential target for the development of new anti-tumour drugs because inhibition of tumour lactate production would decrease tumorigenesis by both decreasing their lethal effects over normal adjacent tissues or augmenting the immunological response against tumour cells [62,63]. These authors concluded that high lactic acid concentrations in the tumour environment block lactic acid export in T-cells, thereby disturbing their metabolism and function.

These findings suggest that targeting this metabolic pathway in tumours is a promising strategy to enhance tumour immunogenicity [63], which was supported recently by Idorn and Hojman [64] proposing a complementary anticancer therapeutic approach based on the effect of NK cell mobilisation and activation, as well as changes of blood perfusion and body core temperature by exercise. These effects may reinforce immune cell distribution and transmigration into tumours, facilitating tumour lysis.

Several treatment strategies, such as glucose deprivation, inhibition of the glycolytic pathway, the use of glucose analogues, and inhibition of the glucose transport, to exploit HIF-1 and to exploit acidosis have been described in detail with respect to the tumour metabolism and the Warburg effect by Gatenby and Gillies [28] and others [37]. As an attractive therapeutic approach, Gatenby and Gillies [28] suggested the inhibition of the glycolytic pathway. They based their assumptions on the fact that cancer cells rely on anaerobic metabolism to produce a variable but generally significant portion of their energy requirements. From this, the inhibition of the glycolytic pathway may be an obvious approach to utilize the high glucose consumption by cancer cells. From several papers [37,56], there is clear evidence that the inhibition of glycolysis can result in cancer cell death due to ATP depletion, particularly in a hypoxic environment.

Wu et al. [65] reported that lactic acidosis was a potent regulator of cancer cell glycolysis. In the absence of lactic acidosis, cancer cells exhibited excessive glycolysis and produced large amounts of lactate. In the presence of lactic acidosis, cancer cells exhibited low glycolytic rates and produced negligible amounts of lactate. However, when culture conditions changed from lactic acidosis to a regular culture, cancer cells instantly switched back to the glycolytic Warburg phenotype, indicating the need to combine treatments. Anderson et al. [66] found that hexokinase-2 promotes tumour growth and metastasis by regulating lactate production in pancreatic cancer. These authors suggested that glycolysis may be important for metastasis. They found that hexokinase-2 directly promoted metastasis via regulation of glycolysis and a pharmacologic inhibition of lactate production prevented the hexokinase-2-driven invasion. This was in line with previous studies showing that extracellular lactate enhanced migration in breast cancer cell lines, encouraged metastasis seeding of breast cancer cell lines in vivo, and promoted mobility of glioblastoma cell lines. These authors suggested that inhibition of either hexokinase-2 or lactate may improve patient outcome by limiting the formation of metastases. The results highlight the need to understand the effects of the site of lactate production, the interplay of tumour, microenvironment, and whole-body systems, as well as the direction of the lactate gradient [66].

The contribution of lactate to the tumour micro-environment and an impaired cytolytic function of T-cells in vitro have been presented recently by Romero-Garcia et al. [51]. These authors discussed lactate secreted by the tumour as an immune-suppressor molecule that contributes to tumour evasion, and possible treatments targeting lactate metabolism were proposed. However, Gatenby and Gillies [28] highlighted that several components of the glycolytic pathway have been targeted for therapy development, although relatively few have investigated in vivo experiments, and even fewer in clinical trials. From their point of view, the most obvious therapeutic target in the glycolytic pathways was hexokinase, which catalyses phosphorylation of glucose to glucose-6-phosphate, the first and rate-limiting step in glucose metabolism. Several papers showed that the lactate analogue 3-bromopyruvate (3BP), a potent hexokinase inhibitor markedly reduced glucose entry into the cytoplasm, rapidly killed cancer cells growing in tissue culture, eradicated tumours in animals, and prevented metastasis. In addition, the substance was suggested as an effective anti-liver cancer agent in humans and was also effective against multiple myeloma. 3BP was shown to significantly extend the life of a human patient for which no other options were available [67,68,69].

However, no clinical trials using this strategy have yet been reported. Other substances such as lonidamine decrease glycolysis in vitro and in vivo, probably through inhibition of mitochondrial-bound hexokinase [70]. This substance was shown to decrease intracellular ATP and lactate production in cancer cells but appears to enhance aerobic glycolysis in non-transformed cells. Chen et al. [71] highlighted the challenge in metabolic anticancer therapy to the development of pharmaceutical agents that could selectively target cancer cells. A better understanding of the biology and the regulatory mechanisms of aerobic glycolysis was suggested to facilitate the development of glycolysis-based therapeutic interventions for cancer. Glycolysis inhibition, combined with DNA-damaging drugs or chemotherapeutic agents, were proposed to be effective anticancer strategies through weakening cell damage repair capacity and enhancing drug cytotoxicity [71].

## 4. Exploiting Acidosis

A consequence of increased glycolysis is acidification of the extra-cellular space [25]. It has been proposed that this perturbation selects for resistance to acid-mediated toxicity, and that these enhance tumour growth and invasion. It also represents an opportunity for therapy because the tumour microenvironment is typically extremely acidic, which, in combination with hypoxia, may produce significant cellular stress leading to intra-tumoural regions of necrosis. Strategies to enhance this toxicity and extend the regions of necrosis stem from recognition that acid escapes from tumours by only two mechanisms: venous efflux and local diffusion. Using mathematical models, Gatenby and Gawlinski [72] demonstrated that venous outflow can be significantly inhibited using even mild systemic acidosis. Briefly, this is because acid flow into intra-tumoural blood vessels is in part dependent on the concentration gradient between the interstitial space and the blood. If the acid concentration in the blood is increased, vascular outflow of acid from the tumour dramatically decreases and the intra-tumoural pH declines precipitously. As a result, local acid concentrations exceed the tolerance of even the tumour cells, triggering extensive tumour necrosis. These theoretical and somehow paradoxical results have not been explicitly tested, but some indirect results are supportive. Harguindey et al. [73] demonstrated tumour-bearing rats fed acidic chow had a substantial survival advantage when compared with control. These authors concluded that total body acidification was promising in antagonizing tumour growth, and its effects were independent of starvation-ketosis. Acidosis was not suggested to favour solid tumour growth, and it was shown to have the potential to delay it, at least in some cases. Similar results were presented in 1932 by Parentjev et al. [74], who applied sodium lactate injections in rats with transplanted tumours. The solution was injected subcutaneously for three weeks into 34 rats with transplanted sarcoma 39, each animal receiving one dose daily. At the end of the experiment, the tumours had disappeared in 16 cases (47%), compared with 23% in the control group. The authors concluded that an increase in the level of lactate in the organism produced by the injection of sodium lactate inhibited the growth of transplanted rat sarcoma and was able to produce a complete disappearance in a certain number of cases [74].

Additionally, Gatenby et al. [72] found that patients who develop renal failure (which is typically associated with metabolic acidosis) after cytoreductive nephrectomy for metastatic renal cancer had a significant survival advantage compared with those who maintained normal postoperative renal function (survival of 17 months vs. four months). Kelley et al. [75] used isolated limb perfusion in a rat model to examine simultaneous perfusion of acid and melphalan for treating melanoma. They found that the combination resulted in consistent cure of the animals, and in addition, they showed that acid perfusion alone (pH of 6.8 for 10 min) induced extensive apoptosis in the tumours and a significant survival benefit. Nakao et al. [76] showed that patients with chronic renal failure had a markedly reduced ability to form lactate with anaerobic exercise; they suggested that this was due to the inhibition of glycolytic enzymes of skeletal muscle by systemic acidosis. Sutton et al. [77] showed that muscle lactate increased most from alkalosis during high-intensity exercise and least with acidosis. The lower plasma lactate concentration during exercise in acidosis was suggested to be due to an inhibition of muscle glycolysis, combined with a reduction in lactate efflux from muscle.

In a recent study on lactate’s effect on human neuroblastoma cell bioenergetic fluxes, Lezi et al. [78] found acute effects of lactate on cell respiration with a dose-dependent increase in oxidation rate and a significant reduction in glycolysis rate following lactate introduction, but glycolytic flux rapidly returned to baseline when lactate was removed. Lactate induced a reversal of the Warburg metabolism and reduced the activation state of the pro-growth insulin-signalling pathway. These authors proposed that bioenergetic intermediates such as lactate can be used to alter bioenergetic fluxes that are assumed to alter the function of proteins that monitor and regulate those fluxes, which in turn change gene expression, inducing relatively durable intracellular changes that persist even after the levels of flux intermediates returned to baseline [78].

From these papers, the importance of the possible impact of glycolysis inhibition may be deduced. Interestingly, this topic has also been investigated in exercise physiology for several years. Therefore, from this point of view, one may suggest therapeutic options from applying regular high-intensity glycolytic exercise training.

On the other hand, a recent study on possible preventive effects of voluntary wheel running on primary tumour growth and metastases formation analysed spontaneous pulmonary metastasis after orthotopic injection of 4T1 breast cancer cells into mammary fat pads of female Balb/C mice [79]. This study identified that, in mice running on wheels, the volume and size of the primary tumour were not affected, but the number of secondary nodules formed in the lungs was significantly increased compared to sedentary counterparts. The authors concluded that voluntary wheel running appeared to impair rather than improve endothelial function and to promote but not decrease metastasis in the murine orthotopic model of metastatic breast cancer. From these results, we carefully need to address the notion of a persistent beneficial effect of voluntary exercise on cancer progression, and pro-metastatic effects of voluntary exercise need to also be expected [79]. In contrast, Higgins et al. [80] showed that Lung tumours in exercising mice grew significantly more slowly relative to sedentary mice, and no mouse developed distant disease, in line with most of the studies showing beneficial effects.

## 5. Definition of High-Intensity Exercise

**Figure 1:**
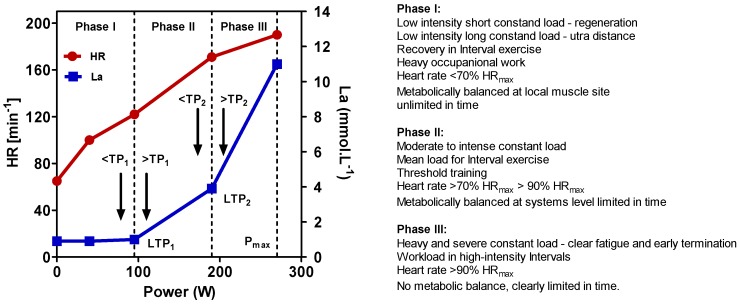
Heart rate (HR) and blood lactate concentration (La) during an incremental ergometer test. Two turn points for La (LTP_1_, LTP_2_) can be determined, discerning the curve into three metabolically and cardio-respiratory different phases as indicated (P_max_ = maximal power output) (adapted from Reference [88]).

**Figure 2:**
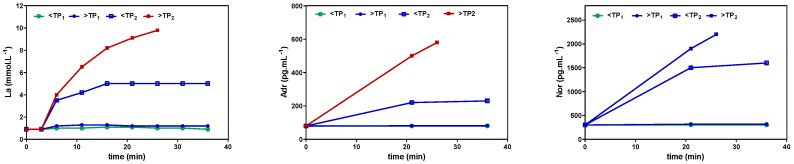
Time course of blood lactate adrenalin (Adr) and noradrenalin (Nor) concentration during constant load exercise just below and above the first (LTP_1_) and the second (LTP_2_) lactate turn points (adapted from Reference [88]).

To define exercise, it is important to apply physiological principles. In line with other authors [81], we recently presented a physiological framework to differentiate high-intensity constant load and intermittent exercise applying the lactate shuttle theory [82,83,84]. Three zones of exercise intensity can be prescribed, which are separated by two thresholds such as the first and the second turn points for ventilation (VT_1_/VT_2_) or lactate (LT_1_/LT_2_) (Figure 1) [81,82], allowing us to individually prescribe exercise intensity for continuous and intermittent-type exercise [83,84,85]. Several markers such as heart rate (HR), power output (P), or oxygen uptake (VO_2_) may be applied to prescribe and regulate exercise intensity [86] (p. 82). It is important to note that the catecholamine response, as shown in Figure 2, presented a similar pattern to lactate, applying different exercise intensities in continuous and intermittent-type exercise. No increase was found below the first threshold, a small increase but constant with time between both thresholds, and a steady increase above the second threshold with early termination due to metabolic acidosis [87,88]. This may be specifically important, as Pedersen et al. [18] showed that epinephrine mobilizes NK cells in a mice model and ß-adrenergic blockade blunted the training-dependent tumour inhibition. We may therefore prescribe exercise intensity to be low if below VT_1_/LT_1_, moderate between VT_1_/LT_1_ and VT_2_/LT_2_, and high above VT_2_/LT_2_ for both continuous and interval exercise [81,82,83,84,85,87,88].

High-intensity exercise may be therefore defined as exercise intensity above the second threshold [83]. This intensity ranges to maximal power output from an incremental test and even up to sprint exercise [89,90]. One key feature of high-intensity anaerobic exercise is its unbalanced metabolic disturbances [83]. It needs to be mentioned that METs usually applied to prescribe workload do not represent the individual strain for a given workload, and we need to be careful to apply the METs concept if the individual response is a main target [91].

As highlighted by Jones et al. [19], current exercise therapy prescriptions in the oncology setting still adopt a one-size-fits-all approach, and they conclude that because exercise therapy is becoming an increasingly important strategy in cancer therapy, an individualized dosing of exercise is necessary to fulfil the requirements for an optimal medical treatment. These authors suggest a variable approach, applying intense doses for initial tumour control and variable and lower doses for maintenance. In line with Jones et al. [19], it has to be mentioned that exercise intensity constitutes the workload, and exercise duration has to be respected beside other variables such as the frequency and the type of exercise. We recently showed that exercise duration has to be included in exercise prescription; however, the identification of the optimum duration is still a challenge [92,93]. Although it is difficult to obtain the maximal duration necessary for exercise duration prescription in patients [93], we may define four zones of load with respect to the maximum possible duration for any single intensity. Going to the maximum duration will lead to severe fatigue, which is necessary to force an optimal adaptation (if necessary recovery is given). Applying a submaximal duration with the same intensity (e.g., 70% of the maximal sustainable duration) will result in reduced fatigue and hence minor adaptation. Reducing duration to approximately half of the maximal duration will give no fatigue, and exercise performance will be maintained without further gains in power but possibly gaining some minor effects in exercise capacity. Reducing duration down to as low as 20% of the maximal duration will affect recovery and will have no effect on power or capacity. Therefore, talking about high-intensity exercise must not necessarily imply high workload if the duration remains short. This may be important, specifically for patients who may benefit from high-intensity metabolic changes in lactate and catecholamine levels but who must not be forced to fatigue by an exhaustive duration. In line with this assumption of acute effects of exercise, Dethlefsen et al. [13,17] proposed from a breast cancer cell viability study that beside the well-known overall accumulative effects of physical activity and exercise training, the effect of each single repeated exercise bout drives the protective effect of exercise on breast cancer outcome. They suggested that the dramatic increase in acute exercise-induced factors such as catecholamine, cytokine, lactate, and immune cells are more important than the moderate decreases in basal levels of risk factors such as insulin, sex hormones, cytokines, and cholesterol [13]. Therefore, these authors highlighted the need for studies on systemic changes occurring during acute exercise, aiming to identify potential exercise-induced anti-oncogenic factors and how these are regulated by different modes of exercise [13,17].

## 6. Inhibition of Glycolysis by High-Intensity Exercise and Lactate Metabolism

Hollidge-Horvat et al. [52] and Parolin et al. [94] repeated maximal sprint exercise. It was shown by these authors that lactate, pyruvate, and H^+^ increased progressively during the first bout of exercise; however, during the consecutive bouts of exercise, lactate, pyruvate, and H^+^ remained elevated but did not increase further. These authors pointed out that the elevated H^+^ and lactate concentration throughout the following bouts of exercise may have completely inhibited the glycogenolytic flux. This mechanism of regulation may have served to spare the glycogen stores, but more importantly, inhibited further accumulation of lactate and increase in H^+^. Thus, the increase in H^+^ that accompanies intense exercise may be the primary mechanism responsible for limiting further increases in H^+^ and the accumulation of lactate while increasing the oxidation of lactate [94].

**Figure 3:**
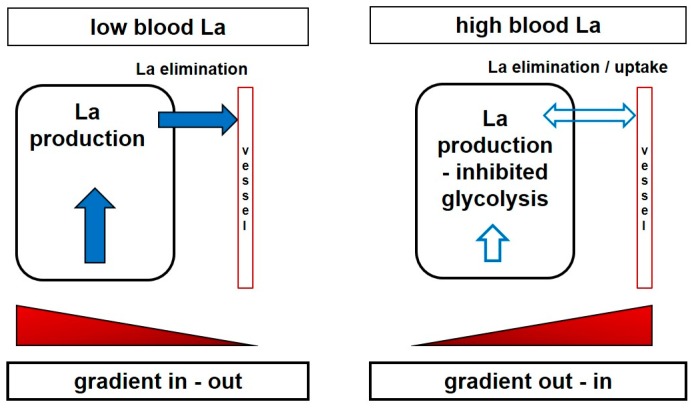
Principle of the relationship between lactate production, systemic blood lactate concentration, and flux direction along a gradient. With a low blood La concentration, cells producing La are able to shuttle La to the system along an outward gradient (**Left**). Increasing La concentration in blood with anaerobic muscular exercise reverses the gradient, and cells trying to produce La are limited to shuttle La against the gradient, inducing intracellular acidification and subsequent inhibition of La production (**Right**).

It may be assumed that the working muscle itself is affected by these changes of the extracellular and/or intracellular milieu. Interestingly, even in the case that the systemic lactate increase is augmented by a muscle group different from the investigated one, Bohnert et al. [95] showed that the increase in lactate and the decrease in pH was lower in a subsequent bout of leg exercise if the pre-loading exercise was performed with an arm exercise that induced a systemic acidosis. Similar results were presented by Bogdanis et al. [96], who showed that the rate of blood lactate accumulation was decreased by approximately 50% when blood lactate levels were pre-elevated by an arm crank exercise, and more pronounced decreases in the rate of blood lactate accumulation were observed after a second sprint, where the delta lactate values were approximately 30% of those found following the first sprint. Both a reduced La-/H^+^ efflux from the muscle and an inhibition of glycolysis by H^+^ were put forward as a possible explanation for the decreased rate of lactate accumulation by these authors. A reduced lactate and H^+^ gradient between muscle and blood, together with a decreased blood and inactive muscle buffering capacity, are probable mechanisms that may also be applied to tumour metabolism (Figure 3). Similar results were presented by Purge et al. [53] in rowers where net lactate increase was substantially reduced with just minor changes in performance.

We may therefore hypothetically suggest that the systemic increase of blood lactate concentration by one or several repeated short and high-intensity exercise bouts inhibits glycolysis in the working muscle itself and in other cells and tissues such as tumour tissue. Smallbone et al. [54] showed the effects of single anaerobic exercise bouts on carcinogen. Model simulations demonstrated that repeated episodes of transient systemic acidosis are able to interrupt critical evolutionary steps in the later stages of carcinogenesis, resulting in substantial delay in the evolution to the invasive phenotype. These results suggest that a transient systemic acidosis may mediate a reduction in cancer risk associated with increased high-intensity anaerobic physical activity. In addition, Dethlefsen et al. [13,17] have shown that each single acute exercise bout may mediate positive effects on breast cancer progression due to systemic factors such as catecholamines and myokines. This side-effect-free intervention, which may selectively influence tumour metabolism by inhibition of glycolysis, seems promising; however, to ascertain possible therapeutic effects, well-structured clinical studies are needed to investigate tumour metabolism before and after high-intensity exercise by means of functional magnetic resonance imaging (fMRI) studies and tissue biopsies [97].

Thus, this well-known effect in high-intensity exercise in exercise physiology as described above may be applied to the intentions to effectively inhibit glycolysis without harmful side effects, by increasing the systemic level of lactate by high-intensity exercise (anaerobic work bouts) [53,95,96] or by means of lactate infusions [98,99], lactate injections [74], or lactate-containing fluid drinks [100]. However, high-intensity anaerobic exercise has rarely been applied in cancer patients, and exercise interventions including single short high-intensity anaerobic bouts of 20–40 s of all-out exercise or any other exercise above VT_2_/LT_2_ increasing whole body lactate concentration are warranted.

## 7. High-Intensity Anaerobic Exercise as a Potential Cancer Therapy?

Usually, moderate-to-vigorous intensity aerobic exercise (if any) is recommended for most cancer patients [7]. Hayes et al. [6] have critically mentioned this and argue that higher-intensity exercise may be needed to reveal significant effects on risk reduction, survival, and recurrence. Jones et al. [19] stated exercise to be a cornerstone intervention for metabolic control and provided a framework for prescription in exercise-oncology research [101]. Exercise was reported to be associated with improvement in metabolic control in patients with breast cancer. Because altered tumour-cell metabolism was strongly linked with cancer, the modifying effects of exercise on metabolic control in patients with cancer, either alone or in conjunction with drug therapy, were argued as an exciting avenue for future investigations [102]. As the impact of exercise is largely influenced by exercise intensity, it may be speculated that higher-intensity exercise training within anaerobic Phase III or even higher (Figure 1) may also be applied in cancer patients. However, papers about such high intensity exercise in cancer patients are still sparse.

A few studies investigated the influence of high intensity applications in cancer patients [103,104]. These papers did not show adverse effects, and the multimodal intensive resistance and aerobic training were well tolerated by patients suffering from various forms of cancer and presenting various stages of disease. The studies are in good agreement for increases in muscular strength, aerobic performance, physical and functional activity, and emotional wellbeing, but not quality of life. However, survival time and disease-free time were not investigated in these studies.

Van Blarigan et al. [105] reported that men who walked briskly after diagnosis had a 48% decreased risk of prostate cancer recurrence compared with men who walked at an easy pace, and brisk walking induced more normal-shaped blood vessels in the prostate tumours, suggesting a greater impact on tumour vasculature by brisk walking. In a recent study by Adams et al. [106], participants completed four high-intensity intervals lasting four minutes each during the workouts, which progressed from 75 to 95% of VO_2peak_ during the intervention period. The high-intensity intervals were separated by 3-min of active recovery intervals performed 5–10% below the ventilatory threshold. These authors provided the first evidence that 12 weeks of high-intensity interval improved cardio-respiratory fitness, multiple pathways of cardiovascular disease risk, and surrogate markers of mortality in testicular cancer survivors. This training significantly improved maximal oxygen uptake, which was suggested clinically relevant, as these changes are likely to reduce overall mortality by 10–25%. Dolan et al. [107], who investigated postmenopausal breast cancer survivors, presented similar results. Patients were randomized into three groups with either supervised aerobic interval training (AIT: 70 to 100% VO_2peak_), supervised continuous moderate exercise training (CMT: 60 to 70% VO_2peak_), or an unsupervised control group (CON) for six weeks. Participants completed the study with no adverse advents. Compared with CON, cardio-respiratory fitness improved significantly in AIT and CMT by 12%, with no significant difference between exercise groups. The authors concluded that the study provided evidence that, similarly to CMT, AIT can safely increase VO_2peak_ in a small group of breast cancer survivors. In line were the results by Edvardsen et al. [108], who conducted a single-blind randomized controlled trial with high-intensity endurance and strength training (three times per week for 20 weeks), starting five to seven weeks after lung surgery. Compared with the control group receiving standard postoperative care, the intervention group performed high-intensity training. The exercise group had a greater increase in peak oxygen uptake, strength, chair stand, stair run, and total muscle mass compared with controls. QoL (SF-36) physical component summary score and mental component summary score significantly improved in the exercise. High-intensity endurance and strength training was well tolerated in these patients recently operated for lung cancer and induced clinically significant improvements in functional fitness and QoL. Devin et al. [109] presented data on colorectal cancer survivors, highlighting that following diagnosis and anti-cancer therapy, cardio-respiratory fitness and body composition declines led to significant increases in morbidity and mortality. As there is increasing interest within the field of exercise oncology regarding the potential strategies to influence adverse outcomes, the authors compared four weeks of moderate-intensity exercise (MIE: 70% HR_peak_) vs. high-intensity exercise (HIE: 85–95% HR_peak_) training on peak oxygen consumption (VO_2peak_) and body composition in colorectal cancer survivors. The high-intensity exercise programme was superior to the moderate one in improving absolute and relative VO_2peak_. The authors concluded that high-intensity training was a safe, feasible and efficacious intervention that offered clinically meaningful improvements in cardio-respiratory fitness and body composition for colorectal cancer survivors. They mentioned that HIE appears to offer superior improvements compared with current physical activity recommendations for colorectal cancer survivors and may therefore be an effective clinical utility following treatment.

Midtgaard [110] showed similar results in a larger randomized controlled study. A 12-month exercise-based rehabilitation programme was an effective strategy to promote physical activity and to improve VO_2peak_ in cancer survivors in a programme consisting of individual (×3) and group-based (×6) counselling in combination with once-weekly high-intensity group-based exercise training.

In another study by Schmitt et al. [111], 28 women who had been treated for breast cancer were randomly assigned to three weeks of low-to-moderate intensity or high-intensity interval training. Work economy improved following both interventions, with improved peak oxygen uptake following low-intensity training. This randomized controlled study demonstrated that female cancer survivors could perform high-intensity interval training without adverse health effects. Since the outcomes were similar, the more time-efficient high-intensity interval training was suggested as a better strategy for improving the health of female cancer survivors.

Kampshoff et al. [112] additionally presented a randomized controlled trial of the effects of high-intensity and low-to-moderate intensity exercise on physical fitness and fatigue in cancer survivors. The exercise programs included six resistance exercises targeting large muscle groups with a frequency of two sets of 10 repetitions and workload defined by one-repetition maximum (1-RM) measurement. The high-intensity group (HI) started resistance exercises in the first week at 70% of 1-RM and gradually increased to 85% of 1-RM in week 12. The low-to-moderate intensity group (LMI) started resistance exercises at 40% of 1-RM and gradually increased to 55% of 1-RM. Two types of endurance interval exercises, aiming to maximize improvements in cardio-respiratory fitness were applied. Patients cycled 2 × 8 min with alternating workloads in the first 4 weeks. Workloads were defined by a maximum short exercise capacity (MSEC) obtained by a steep ramp test. The HI group cycled 30 s at a workload of 65% of the MSEC and 60 s at 30%, and the LMI group cycled 30 s at a workload of 45% of the MSEC and 60 s at 30%. In both groups, 74% and 70% of the participants attended more than 80% of the prescribed exercise sessions. Both training programmes exhibited significantly (*p* < 0.05) larger improvements in VO_2peak_ compared with wait list controls, but the improvements were larger for high-intensity exercise, although not statistically significant (*p* = 0.08). Exercise significantly reduced general and physical fatigue and global quality of life and anxiety after high-intensity exercise. The authors concluded that shortly after completion of cancer treatment, both high- and low-intensity exercise were safe and effective, but there may be a dose–response relationship between exercise intensity and VO_2peak_, favouring high-intensity exercise. Unfortunately, the prescription of exercise in this study does not allow to clearly relate the physiological response to a specific intensity zone.

These results are in line with results by Martin et al. [113] showing that higher-intensity exercise provided more sustainable cardio-respiratory benefits than lower-intensity exercise. These authors suggested that survivors need guidance on exercise intensity, because a high volume of low-intensity exercise may not provide sustained health benefits.

In a recent study adding high-intensity interval training to conventional training during chemotherapy in breast cancer patients, Mijwel et al. [114] showed that 16 weeks of resistance and high-intensity interval training were effective at preventing a reduction in cardio-respiratory fitness and reduced symptom burden. Compared to usual care, combined resistance and high-intensity interval training was superior for total symptoms (*p* < 0.01). Subjects performed 2–3 sets of 8–12 repetitions at an intensity of 70% of the estimated 1-repetition maximum (1-RM) and progressed to 80% when the number of repetitions exceeded 12. High-intensity interval exercise was composed of 3 × 3 min bouts of aerobic intervals at a rating of perceived exertion (RPE) of 13–15.

These above-mentioned studies applied high-intensity in part anaerobic endurance or resistance exercise, but the number of studies is still too low and heterogeneous for mode and intensity to draw some final conclusions. Additionally, only overall but not the single bout effects have been studied, although recent studies showed that each single bout response may be a key to understand the beneficial impact of exercise in cancer therapy [13,17].

Some animal studies were found applying high-intensity anaerobic exercise training [115,116]. Anaerobic exercise consisting of six sets of 10 jumps in water with an overload of 50% of body mass with 1 min of rest performed four times per week for eight weeks decreased tumour growth, cancer cachexia (less weight loss), and increased innate and adaptive immune function with increased lymphocytes, phagocytosis and lysosomal volume in tumour-bearing rats [116]. The study presented by Bacurau et al. [115] yielded similar results, and the authors suggested that high-intensity exercise training may be a viable strategy against tumours. There was a reduction in tumour growth, which was associated with an impairment of tumour cell glucose and glutamine metabolism. Exercise training increased the life span of rats 2.8-fold (16 ± 2 days versus 45 ± 5 days), and tumour mass related to whole body mass was significantly reduced by 10% in the trained rats (17.3% vs. 7.0%) [115] or was 34% lower than the control group [116]. The high-intensity training changed the pattern of glucose and glutamine metabolism presented by Walker 256 tumour cells isolated from the animals, whereas exercise reduced lactate production by 72% and glutamine decarboxylation by 98.2%. The metabolic changes were accompanied by a reduction in symptoms associated with cachexia, such as excessive body weight reduction and decrease in food intake [115]. The decreased tumour growth was explained by the metabolic changes induced by high-intensity anaerobic-type exercise training. A significant reduction of serum lactate concentration was found for the trained tumour-bearing rats although this was still higher than in non-tumour-bearing rats. The reduction in tumour growth was accompanied by an increase in lymphocyte proliferation and macrophage function [116].

Paceli et al. [117] investigated the effects of aerobic and anaerobic exercise versus controls on the development and the progression of lung cancer in mice. Anaerobic but not aerobic exercise diminished the incidence of experimental lung tumours, and no metastasis or significant changes in other organs were observed in all groups. Piguet et al. [118] have presented similar results where liver tumours’ development decreased in mice although regular exercise applied was set at moderate aerobic intensity, indicating that even moderate-intensity exercise may be able to induce chronic effects.

Verma et al. [119] investigated the influence of intense treadmill running in mice with induced Dalton´s lymphoma (DL). Physical exercise resulted in a duration-dependent appreciable decrease in the blood vessel distribution around the peritoneal area, along with a decrease in the red blood cell density in the peritoneal fluid compared with DL-bearing mice without exercise. Additionally, exercise reduced vascular endothelial growth factor (VEGF), and the level of VEGF was comparable to control mice without DL. A decline in lactate content was accompanied by an increase in pH to normal values in the DL mice with exercise. The authors concluded from their results that anti-tumour actions of physical exercise could be mediated by interplay of a number of soluble mediators of tumour microenvironment, regulating tumour cell apoptosis and survival. A recent study about high-intensity interval training together with tamoxifen application in mice showed that interval exercise may reduce mammary tumour burden through possible underlying pathway of microRNA 21 [120]. Interesting to note is that the average tumour volume was significantly less in the interval-trained mice compared with control, independent of the additional tamoxifen application [120].

Although there is good agreement on the beneficial effects of the usual moderate intensity exercise to prevent and treat cancer, there is still a lack of high-intensity studies. Additionally, the large inconsistency in the studies regarding the exercise dose with special reference to intensity, duration and frequency, as well as timing and length of treatment hinders compelling conclusions [1]. In line with this article, Jones et al. [19] pointed out that as for healthy subjects [82] and heart disease patients [81], exercise prescription needs to be specific and targeted to the primary endpoint or system(s) or pathways known or postulated to underpin the effects of exercise on the primary therapeutic target. Contrary to conventional treatment, a non-linear prescription of exercise has been recommended, inducing an initial tumour control with intense exercise and maintenance with smaller and variable doses. Unfortunately, no such study has been conducted so far. High-intensity anaerobic exercise, which was shown to be safe and effective in animal studies and patients, may be used as one specific part of such a comprehensive exercise oncology training approach [19], which should be included in cancer rehabilitation programmes [121]. However, we critically need to mention that at the moment we just can conclude that patients can perform high-intensity exercise such as resistance and interval-type exercise safely and beneficial effects have been prescribed [17] although the specific details have not been investigated in much detail yet. It was shown that during a six-month exercise intervention, women diagnosed with operable stage I-III breast cancer were able to perform supervised exercise training consisting of one weekly group-based session comprising aerobic and resistance training for a total of 90 min per session. Aerobic training consisted of high-intensity interval cycle ergometer training with interval-duration and intensity ranging from 30 s at maximum intensity up to 6 min at 80–90% of maximal heart rate and an exercise:recovery ratio of 1:2–3:1. Resistance training consisted of three sets of 8–10 repetitions at 70–90% of the one repetition maximum (1RM) involving six muscle groups [17]. Lactate concentration increased up to approx. 10 mmol·L^−1^ in an experimental session and the serum applied to breast cancer cell lines demonstrated inhibitory effects on breast cancer viability [17]. These authors proposed that the acute transient changes during each exercise bout drive the positive effects of exercise on cancer outcome rather than the systemic adaptations seen with training over time. One of the numerous factors that may have an influence may be the counter-regulatory effect of an elevated systemic lactate level that has not yet been addressed in those studies.

In a recent article by San-Millan and Brooks [40], the authors viewed cancer cell biology from perspective of the lactate shuttle concept, wherein lactate produced at one intracellular site can elicit a host of autocrine, paracrine, and endocrine responses. Because there are so many metabolic characteristics in common between working muscles and cancer cells, these authors think it reasonable to assess whether new knowledge in skeletal muscle metabolism during exercise can be useful to cancer biology researchers. There is some hope that this new perspective will lead to a broad discussion of lactate metabolism in cancer that could lead to the development of new diagnoses and therapeutics. Critically, it has to be mentioned that the argument for the impact of high intensity exercise on the Warburg Effect is not yet based on experimental data directly addressing the hypothesis, although the actual as well as older data hypothetically indicate this prescribed beneficial effect. Given that there are so many other potential mechanistic explanations for the beneficial effects of exercise on cancer risk, tumour biology, rand ecurrence, studies investigating the acute effects of various exercise intensities and modes need to be performed with respect to this multitude of influencing factors and their relationships [5,13].

## 8. Conclusions

Moderate-to-vigorous aerobic as well as resistance exercise were shown to exhibit overall beneficial effects in the prevention and therapy of cancers. Mechanistic studies are sparse, but results point to a direct effect of exercise on most of the hallmarks of cancer. One key element of most cancers is a highly up-regulated glycolytic phenotype inducing local acidosis, which favours tumour development and invasion. Therefore, this key metabolic function was recognized as a therapeutic target, and several drugs have been developed and tested. As high-intensity anaerobic exercise is known to inhibit glycolysis even at distant sites from the lactate-producing muscles, this physiological principle may be applied as a therapy option to counteract tumour glycolysis. Some rare studies have already applied anaerobic exercise, showing a favourable outcome compared to moderate exercise controls. Although these animal and human studies are encouraging, we need to remark that careful adaptations of exercise are necessary with respect to the need and specific limits of each patient, favouring a personalized exercise therapy applying appropriate intensity, duration, frequency, and type of exercise. However, additional high-quality studies are needed on the influence of acute short and non-exhaustive bouts of exercise with selected and defined exercise intensities and durations in order to identify possible therapy options in various cancers and eligible patients.
